# Estimating the real burden of gestational syphilis in Brazil, 2007–2018: a Bayesian modeling study

**DOI:** 10.1016/j.lana.2023.100564

**Published:** 2023-08-01

**Authors:** Guilherme Lopes de Oliveira, Andrêa J.F. Ferreira, Carlos Antônio de S.S. Teles, Enny S. Paixao, Rosemeire Fiaccone, Raquel Lana, Rosana Aquino, Andrey Moreira Cardoso, Maria Auxiliadora Soares, Idália Oliveira dos Santos, Marcos Pereira, Maurício L. Barreto, Maria Yury Ichihara

**Affiliations:** a Centre of Data and Knowledge Integration for Health (CIDACS), Instituto Gonçalo Moniz, Fiocruz, Salvador, Bahia, Brazil; b Department of Computing, Federal Centre of Technological Education of Minas Gerais, Belo Horizonte, Minas Gerais, Brazil; c The Ubuntu Center on Racism, Global Movement, Population and Equity, School of Public Health, Drexel University, Pennsylvania, USA; d London School of Hygiene and Tropical Medicine, London, UK; e Statistics Department, Institute of Mathematics, Federal University of Bahia, Salvador, Bahia, Brazil; f Barcelona Supercomputing Center, Catalonia, Spain; g Institute of Collective Health, Federal University of Bahia, Salvador, Bahia, Brazil; h National School of Public Health, Fiocruz, Rio de Janeiro, Rio de Janeiro, Brazil

**Keywords:** Sexual transmitted infection, Syphilis, Syphilis during pregnancy, Under-registration, Underreporting

## Abstract

**Background:**

Although several studies have estimated gestational syphilis (GS) incidence in several countries, underreporting correction is rarely considered. This study aimed to estimate the level of under-registration and correct the GS incidence rates in the 557 Brazilian microregions.

**Methods:**

Brazilian GS notifications between 2007 and 2018 were obtained from the SINAN-Syphilis system. A cluster analysis was performed to group microregions according to the quality of GS notification. A Bayesian hierarchical Poisson regression model was applied to estimate the reporting probabilities among the clusters and to correct the associated incidence rates.

**Findings:**

We estimate that 45,196 (90%-HPD: 13,299; 79,310) GS cases were underreported in Brazil from 2007 to 2018, representing a coverage of 87.12% (90%-HPD: 79.40%; 95.83%) of registered cases, where HPD stands for the Bayesian highest posterior density credible interval. Underreporting levels differ across the country, with microregions in North and Northeast regions presenting the highest percentage of missed cases. After underreporting correction, Brazil’s estimated GS incidence rate increased from 8.74 to 10.02 per 1000 live births in the same period.

**Interpretation:**

Our findings highlight disparities in the registration level and incidence rate of GS in Brazil, reflecting regional heterogeneity in the quality of syphilis surveillance, access to prenatal care, and childbirth assistance services. This study provides robust evidence to enhance national surveillance systems, guide specific policies for GS detection disease control, and potentially mitigate the harmful consequences of mother-to-child transmission. The methodology might be applied in other regions to correct disease underreporting.

**Funding:**

10.13039/501100003593National Council for Scientific and Technological Development; The Bill Melinda Gates Foundation and 10.13039/100010269Wellcome Trust.

**Research in context**

**Evidence before this study**

The World Health Organization (WHO) estimates that syphilis infection affects one million pregnancies yearly worldwide, with important consequences for pregnant women and newborns. Untreated gestational syphilis (GS) has been linked to a significantly higher incidence of stillbirth, neonatal death, prematurity, and low birth weight. Notifying GS is compulsory for all WHO member countries; it is mandatory to communicate all cases to health authorities and investigate them. However, the lack of quality in the case detection and data recording processes may contribute to under-registration of GS cases on the health information systems, which underestimates the real magnitude of the disease, especially in middle-income countries such as Brazil.

We searched PubMed/Medline, EMBASE, and LILACS using the search strategy (“underreporting of syphilis” OR “syphilis underreporting” OR “under-registration of syphilis” OR “under-registration”) AND (“Pregnant women”) AND (Brazil) seeking for studies on under-registration of GS across Brazil until July 2023. We supplemented the literature review with internet searches (Google scholar and Semantic scholar), analysis of references in identified papers, and the authors’ own knowledge. We found 6 studies focusing on small to medium-sized Brazilian cities, which indicated: 13.74% of GS under-registration between 2010 and 2013 in the west of São Paulo State; 6.5% of GS notification in Montes Claros city, Minas Gerais State, between 2007 and 2013; 50% more cases of congenital syphilis than GS cases from 2009 to 2015 in Londrina, Paraná State; and 46% of GS underreporting in a special indigenous sanitary district in Mato Grosso State. None of these studies covered the whole country and they do not considered factors associated with underreported cases of GS or even provided corrected estimation of GS incidence. Based on such literature review, the modeling of syphilis under-registration is also rare worldwide since none study was found on this regard.

**Added value of this study**

This is the first study to map the quality of GS notifications at the national, regional, and small area levels in a large country such as Brazil, emphasizing that GS detection and incidence are related to socio-economic conditions and access to health services such as deprivation level and adequate prenatal care. States and microregions in the Brazilian North and Northeast regions presented the highest GS underreporting cases. Health managers and policymakers could easily use our methodology to amplify national syphilis surveillance and estimate the real incidence of GS. Also, the statistical modeling strategy may be used as a reference for application in other middle-income countries.

**Implications of all the available evidence**

Taken together, modeling results from this national-level study on GS burden in Brazil, along with findings from other local surveys, suggest that GS remains a significant public health problem, highlighting the need for improved surveillance. Our research reveals important differences in underreporting of GS cases across Brazilian regions, as well as the association of syphilis surveillance quality with socioeconomic conditions and access to healthcare. This underscores the urgent need to enhance syphilis surveillance and case notification within the healthcare system, particularly in the country's poorest region. Furthermore, correcting GS incidence at different area levels could provide a better understanding of the epidemiological profile and help estimate the social, economic, and health burdens associated with the disease nationwide. Additionally, our findings offer valuable insights for policymakers to enhance planning and take action to reduce the burden of GS, thereby contributing to the reduction of mother-to-child transmission and adverse effects during childbirth.

## Introduction

Syphilis is a sexually transmitted infection caused by *Treponema pallidum*, first identified in 1905.^1^ Despite that, it is still a persistent public health issue in many countries worldwide since the rate of syphilis has increased dramatically in recent decades,^1^ becoming both a burden and damage to public health,^2^, ^3^, ^4^ particularly, during pregnancy. Syphilis diagnosed during pregnancy (also called Gestational Syphilis (GS) and hereafter referred to as such) is the second leading cause of stillbirth globally and results in severe adverse outcomes in more than 50% of cases, such as prematurity, low birth weight, miscarriage, stillbirth, and neonatal death.^1^

Data from 2016 shows that over 900,000 pregnant women were diagnosed with syphilis worldwide, resulting in more than 350,000 adverse outcomes, from which over 200,000 were stillbirths or neonatal deaths.^5^^,^^6^ The region of the Americas had the second-highest estimated prevalence of GS worldwide (0.86%), after Africa (1.52%).^6^ In 2016, the Brazilian Ministry of Health declared gestational and congenital syphilis as an epidemic in the country due to the exponential increase in their incidence rates, with unfavorable outcomes for both women and newborns.^7^

Despite the mandatory registration of gestational syphilis and the notable increase in notifications in recent years, underreporting GS cases is still a problem in Brazil.^8^, ^9^, ^10^, ^11^, ^12^ Failures on report GS could bias the actual scenario of the disease and compromise the knowledge of the real burden of GS in the country. Many factors can influence the underreporting (under-registration) of GS cases in Brazil, mainly the inequality of access to health services and prenatal care, failures in syphilis testing during the antenatal period, and failures to register the notification in the Health Information Systems (HIS).^13^, ^14^, ^15^

Accurate reporting of GS cases allows policymakers to design control policies and helps to reduce its consequences for pregnant individuals and their offspring. Although Brazil and other countries recognize the importance of improving the quality of syphilis detection and notification during pregnancy,^16^ epidemiological studies adopting robust methodologies to analyze underreporting of GS are rare. Few studies have addressed this issue in Brazil and most of the available literature presents analyses with small sample sizes and focuses on specific regions.^9^, ^10^, ^11^ Despite those efforts, there are still gaps in the investigation of underreporting of GS across the country and correction of its incidence. This study aims to estimate the real burden of GS in Brazilian microregions from 2007 to 2018 by applying a statistical modeling strategy to correct under-registration of cases in an official data reporting system, which may be used as a reference for application in other middle-income countries. Measures of underreporting levels are provided by comparing the model estimates with the reported data.

## Methods

### Study design and target population

We conducted a cross-sectional ecological study design, considering GS cases notified in Brazil between 2007 and 2018. Brazil, a continental country, divided into 27 states and five macroregions (North, Northeast, South, Southeast and Midwest), comprising 557 microregions, which are defined as contiguous aggregations of the 5570 Brazilian municipalities.^17^

### Data sources and variables description

This study used administrative non-identified data available from public Brazilian databases. The outcome variable, the count of GS cases, was extracted from the Notifiable Diseases Information System (SINAN in Portuguese: *Sistema de Informação de Agravos de Notificação*) using data received from the Brazilian Ministry of Health in 2019. In Brazil, it is mandatory for healthcare professionals to register GS cases through SINAN-Syphilis, where they complete a form including personal, socioeconomic, and gestational information of the patient at the time of diagnosis.^14^

GS cases registered in SINAN-Syphilis followed the definition described by the Brazilian Ministry of Health^14^^,^^18^ considering active detection. This includes cases where a pregnant individual tests positive for the venereal disease research laboratory (VDRL) test with any titration during pregnancy, provided they have not received prior treatment. Additionally, cases may be considered if a confirmatory test (treponemal serology) is also positive. From the total 305,891 GS cases notified in Brazil between 2007 and 2018, we excluded: i) 131 cases for which the municipality of residence was not identified or it did not match the official municipality codes of the Brazilian Institute of Geography and Statistics (IBGE)^17^; and ii) 2 cases registered in Fernando de Noronha, a Brazilian archipelago located more than 500 km away from the Brazilian mainland, which could prevent a spatial analysis with the method considered in our study. Therefore, our analysis was based on 305,758 (99.96%) GS cases notified in SINAN-Syphilis from 2007 to 2018.

For statistical analyses of GS incidence rates and underreporting level in Brazilian microregions, we collected socio-demographic information and access to health service indicators, as well as factors associated to quality of SINAN-Syphilis form registration. The GS incidence rates were calculated per 1000 live births in the microregion. All variables considered in our study are detailed in Table 1.

**Table 1:** Description of variables and data sources used in the study.

Source and Reference	Variable Name	Definition
Outcome Variable		
SINAN-Syphilis	Gestational syphilis (GS) notified cases	GS cases are defined by an active detection through a reagent result for venereal disease research laboratory test (VDRL), with any titration, during any stage of the gestational period, since pregnant were not previously treated, and/or presented a confirmatory test for syphilis (treponemal serology). Cases were aggregated in each of the 557 Brazilian microregions for the period 2007–2018.
**Factors considered in GS incidence rates modeling**		
CIDACS^19^	Brazilian deprivation index (BDI)	Composite socioeconomic measure estimated for all Brazilian municipalities based on income, schooling, and household conditions information from the 2010 Brazilian Census, ranging from less deprived to more deprived areas.^19^ The average BDI of the municipalities was calculated for the microregion level.
Information System on Live Births (in Portuguese: *Sistema de Informações sobre Nascidos Vivos—*SINASC)	Live births	Number of live births reported in Brazilian microregions from 2007 to 2018.
	Adequacy of prenatal care	The proportion of live births reported in Brazilian microregions between 2007 and 2018 whose mothers attended seven or more prenatal appointments.
	Non-white live births	The proportion of non-white live births registered in Brazilian microregions between 2007 and 2018.
**Proxies for quality of GS cases registration**		
Information System on Live Births (in Portuguese: *Sistema de Informações sobre Nascidos Vivos—*SINASC)	Coverage of prenatal care	The proportion of live births reported in Brazilian microregions between 2007 and 2018 whose mother attended at least one prenatal appointment in the same study period.
SINAN-Syphilis	Test acceptability	The proportion of GS notified cases with a qualitative result (reactive or non-reactive) for at least one non-treponemal or treponemal test in each Brazilian microregion from 2007 to 2018.
	Diagnosis opportunity	The proportion of GS notified cases in which the diagnosis was laboratory confirmed during the first or second trimester of pregnancy in each Brazilian microregion from 2007 to 2018.
	Consistency	The proportion of municipalities within the microregion for which the number of registered GS cases is greater or equal to congenital syphilis cases registered from 2007 to 2018.
	Adequacy of case classification	The proportion of GS notified cases from 2007 to 2018 with sufficient information to be classified within one of the three case definitions (primary, secondary, and tertiary or latent) established by the Brazilian Ministry of Health.
Oliveira et al. (2022)^20^	Completeness score of GS care assistance indicators	A completeness score proposed with basis on variables associated with care assistance^20^ (e.g., information on partner’s treatment, treponemal test realization, treatment regime specification, prenatal care attendance, and description of the evolution of case) extracted from the SINAN-Syphilis forms for GS notified cases in the period 2007–2018.

GS = gestational syphilis; SINAN: Notifiable Diseases Information System (SINAN in Portuguese: *Sistema de Informação de Agravos de Notificação*); SINASC: Information System on Live Births (SINASC in Portuguese: *Sistema de Informações sobre Nascidos Vivos*); CIDACS: Centre of Data and Knowledge Integration for Health (CIDACS in Portuguese: *Centro de Integração de Dados e Conhecimentos para Saúde*); BDI: Brazilian Deprivation Index.

### Statistical modeling

In the statistical literature, correction of underreporting in count data has been done based on censored^21^ and compound^22^, ^23^, ^24^, ^25^, ^26^ Poisson approaches. In both cases, due to identifiability issues, the model depends on the availability of extra information to support the data reporting process modeling. Thus, statistical inference is performed under the Bayesian framework, in which the extra information is accommodated through appropriate prior distributions. In this work, we applied the Bayesian hierarchical Poisson model proposed by Oliveira et al. (2022).^24^ Provided that the Brazilian microregions are clustered according to the quality of GS data, this model only requires specification of an informative prior distribution for the reporting level in areas belonging to the best data quality cluster. This requirement is more feasible in our case, as we do not have: (i) validation datasets^25^; (ii) information about global GS reporting level in Brazil^23^; (iii) reliable local active search survey^26^; nor (iv) informative prior for the reporting probability in all groups of areas.^22^

#### Clustering analysis

We applied the K-means method to group the microregions according to their quality of GS data. We considered seven indicators as proxies for GS data quality: coverage of prenatal care; adequacy of prenatal care; test acceptability; diagnosis opportunity; consistency; adequacy of case classification; and completeness score of GS care assistance indicators (see Table 1). Variables were standardized before applying the K-means method in software R,^27^ using packages^28^ and factoextra.^29^ Definition of the possible optimal number K of clusters was based on the elbow method, which relies on evaluating the total within-cluster sums of squares.^30^ A sensitivity analysis was performed considering different values for the number K of clusters around the optimal region identified with the elbow method. In each case, the clusters were hierarchically related from the best to the worst with respect to data quality. To accomplish this, we analyzed the within-cluster centroid, which contained the mean value for the seven variables used in the clustering analysis. The higher the centroid mean, the better the data quality.

#### Model specification

Let $Y_{i}$ be the total reported (observed) GS cases in microregion i, where i=1,...,557. In underreported scenarios, Oliveira et al. (2022)^24^ proposed assuming that

(Eq. 1) $$Y_{i}|\mu_{i},\varepsilon_{i}\;∼Poisson\left(\mu_{i}\varepsilon_{i}\right)\text{,}$$

where parameters, $\mu_{i}>0$ and $0\;{<\;\varepsilon }_{i}<\;1$ denote, respectively, the GS incidence rate and the proportion of the true (unobserved) cases reported in the i-th microregion. We modeled the GS incidence rate $\mu_{i}$ through the following regression structure

(Eq. 2) $$\log\left(\mu_{i}\right)=\log\left(P_{i}\right)+\beta_{0}+\beta_{1}X_{1i}+\beta_{2}X_{2i}+\beta_{3}X_{3i}+\varphi_{i}\;+\;\delta_{i}\text{,}$$

where $P_{i}$ = total live births (offset), $X_{1i}$ = Brazilian deprivation index, $X_{2i}$ = proportion of live births with adequate prenatal care, $X_{3i}$ = proportion of non-white live births (see descriptions in Table 1), and *log* represents the natural logarithm function. Those three covariates were selected on the basis of literature review, in which it is known that socio-demographic and adequacy to health care indicators tends to be related to the disease incidence.^16^^,^^31^, ^32^, ^33^ They were available in our data sources and then were used to account for discrepancies on socio-demographic and adequacy to health care among the Brazilian microregions. Variables $X_{1}$, $X_{2}$ and $X_{3}$ were standardized before applying the model to estimate their respective effects (parameters) $\beta_{0},\beta_{1},\beta_{2}$ and $\beta_{3}$. Terms $\delta_{i}$ and $\varphi_{i}$ are, respectively, random effects accounting for residual overdispersion induced by local and spatial variations. As it is usual in spatial statistics modeling, the prior distribution for the spatially structured random effect $\varphi_{i}$ is represented by an intrinsic conditional auto-regressive (ICAR) model and $\delta_{i}$ follows a non-informative Gaussian prior.^34^

Following Oliveira et al. (2022),^24^ we assume that $\varepsilon_{i}=1-\gamma_{1}$ for all areas belonging to the best data quality group, $\varepsilon_{i}=1-\gamma_{1}-\gamma_{2}$ for all areas belonging to the second best data quality group, and so on, implying that $\varepsilon_{i}=1-\gamma_{1}-\gamma_{2}-\ldots -\gamma_{K}$ for all areas within the worse data quality cluster. Parameter $\gamma_{1}$ represent the proportion of underreported cases in areas classified in the highest level of data quality; $\gamma_{2}$ is the increment on such proportion for areas experiencing the second highest data quality level, and so on. Such parameters are hierarchically correlated through an appropriate conditional Uniform prior distribution.

The most important and attractive feature of this modeling strategy is that, to attain identifiability, it only requires an informative prior distribution for parameter $\gamma_{1}$, which represents the proportion of underreporting in those areas belonging to the best data quality cluster. An informative prior means a distribution that is highly concentrated in a specific subset of the parametric space. In our application to Brazilian GS data, with basis on literature review and information collected from experts’ on the study of syphilis burden in Brazil, we performed a sensitivity analysis considering four different prior distributions for $\gamma_{1}∼Uniform\left(0,0.02\right)$, $\gamma_{1}∼Uniform\left(0,0.05\right)$, $\gamma_{1}∼Uniform\left(0.05,0.10\right)$ and $\gamma_{1}∼Uniform\left(0.05,0.10\right)$. Respectively, these priors imply that areas classified in the highest level of data quality report, on average, 99.0%, 97.5%, 95.0% and 92.5% of the GS cases. All these priors impose that less than 10.0% of the true GS cases are missed in such areas. Non-informative priors are elicited for parameters $\gamma_{2},\ldots ,\gamma_{K}$.

By varying the number of clusters K and the prior distribution for parameter $\gamma_{1}$, we fitted 12 different models. The WAIC^35^ and the LPML^36^ goodness-of-fit metrics were considered for model selection. For the WAIC, the smaller the value, the better the model fitted the data. Larger values of LPML indicate better fit. A more detailed discussion is presented in Section 1 of the Supplementary File, which includes the prior specification for all parameters of our Bayesian model as well as particularities of model implementation and validation.

It worth mentioning that, for each microregion, provided that $\mu_{i}$ and $\varepsilon_{i}$ are estimated, the underreported GS cases, denoted here by $Z_{i}$, can be estimated from the predictive posterior distribution $Z_{i}|\mu_{i},\varepsilon_{i}\;∼Poisson\left(\mu_{i}\left(1-\varepsilon_{i}\right)\right)$. Then, the total GS cases can be estimated/predicted as $T_{i}=Y_{i}+Z_{i}$, in such a way that the ratio between $Y_{i}$ and $T_{i}$ provides a coverage for the SINAN-Syphilis. The Bayesian highest posterior density credible interval with level 90% was used to evaluate significance of covariates $X_{1}$, $X_{2}$ and $X_{3}$ and to quantify uncertainty in the model predictions.

### Ethics approval

Not applicable. All analyses used de-identified and publicly available data.

### Role of the funding source

The study’s funders had no role in the study design, data collection, data analysis, interpretation, or writing of the manuscript.

## Results

**Fig. 1:**
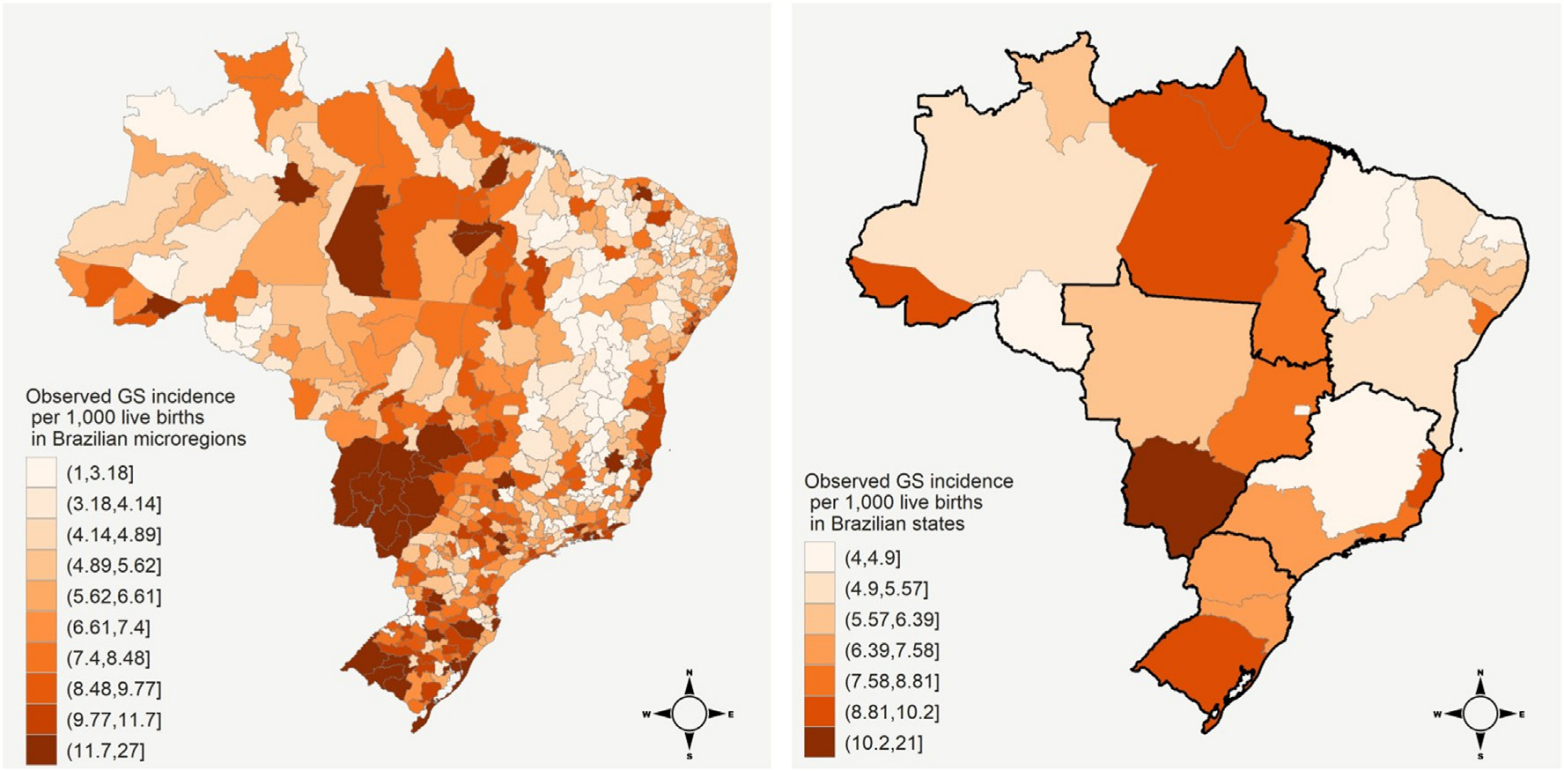
Incidence rates of gestational syphilis (per 1000 live births) in the 557 Brazilian microregions (left) and the 27 Brazilian states (right), 2007–2018, according to the observed data. Brazilian regions (North, Northeast, Midwest, Southeast, and South) are separated by black lines on the right map.

From January 2007 to December 2018, 305,758 GS cases were registered in Brazil corresponding to a GS incidence rate of 8.74 per 1000 live births. GS incidence rates vary across the Brazilian territory (Fig. 1), where the highest observed incidence rate at microregion and state levels were, respectively, 23.45 and 18.12, while the lowest ones were around 1.01 and 4.07, respectively. Northeast and North regions generally presented low incidence rates, as well as most microregions within Minas Gerais State (in Southeast region). Large clusters of microregions with high GS incidence rates were observed in states of Rio Grande do Sul (South region) and Mato Grosso do Sul (Midwest region), which is the only with an incidence rate above 10.2.

**Fig. 2:**
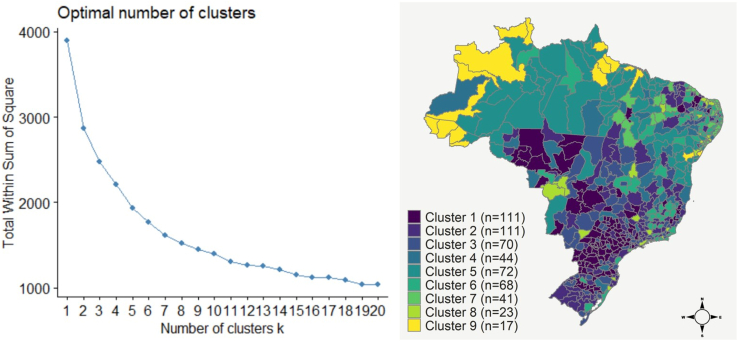
Result of clustering analysis for quality of gestational syphilis reporting in Brazil, 2007–2018. Left: Plot of within cluster sum of squares versus the number of clusters K, indicating that around K = 10 is a good choice in this case according to the elbow method. Right: Map of the 557 Brazilian microregions according to the data reporting quality clusters obtained through the K-means method with K = 9, the best fit for the gestational syphilis database.

To correct for GS underreporting, we performed a sensitivity analysis by applying the Bayesian model with 12 different specifications, which are related to the combinations between the number of clusters (K) and the prior distribution elicited to parameter $\gamma_{1}$ (Supplementary Table S1 of the Supplementary File). The elbow method indicated that K = 9, K = 10 or K = 11 would be a good choice for the number of clusters (Fig. 2). According to the model evaluation metrics (Supplementary Table S1 of the Supplementary File), K = 9 was the best choice to aggregate the 557 Brazilian microregions according to the seven selected proxies of GS data quality. Data quality decreases from Cluster 1 (best data quality group) to Cluster 9 (worst data quality group) (Fig. 2). All the 17 microregions within Cluster 9 are located in the North (14 areas) and Northeast (3 areas) regions.

**Fig. 3:**
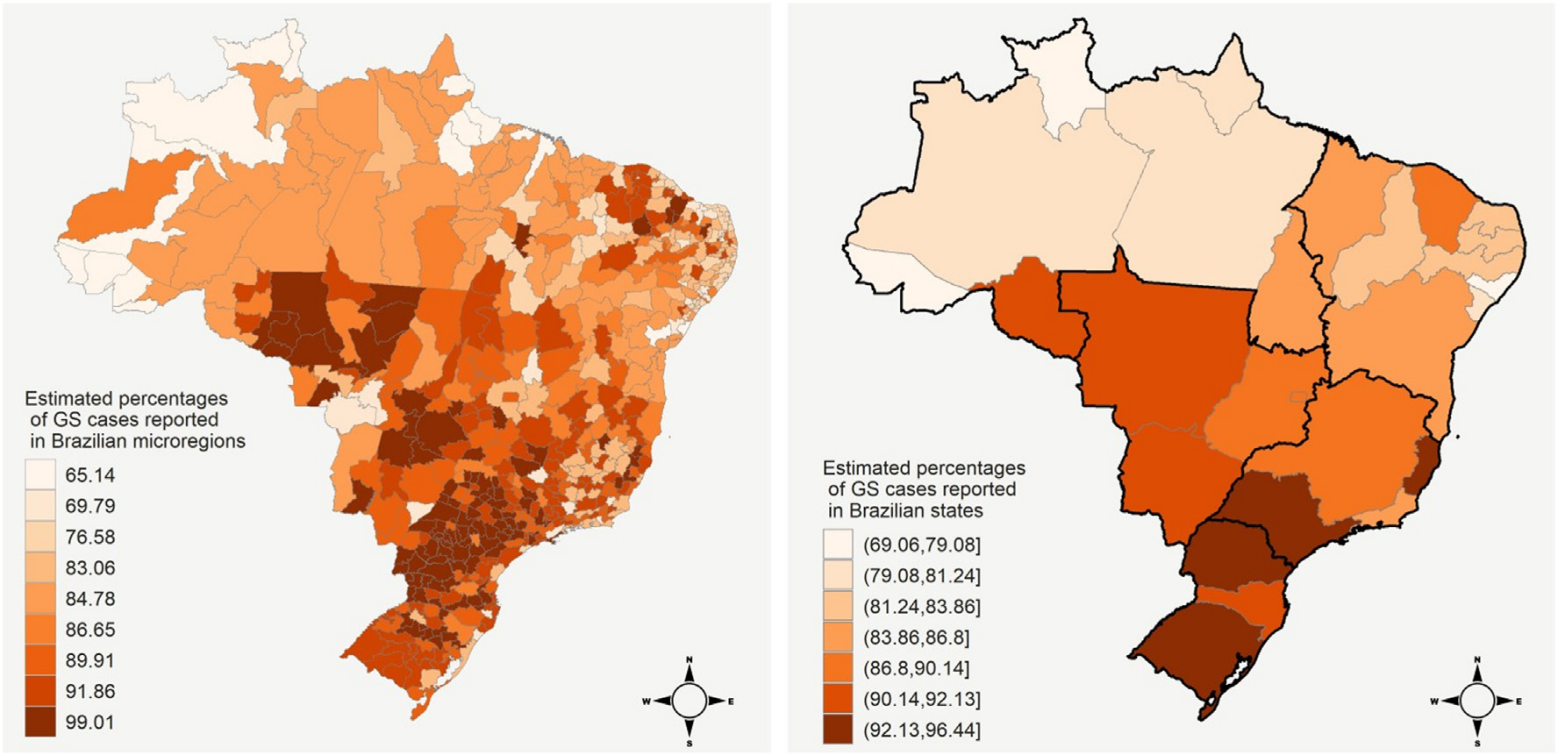
Posterior mean for percentages of GS cases reported in the 557 Brazilian microregions (left) and in the 27 Brazilian states (right) provided by the fitted Bayesian model. Brazilian regions (North, Northeast, Midwest, Southeast and South) are separated by black lines in the right map.

The sensitivity analysis indicated that, *a priori*, the proportion of underreported GS cases in the best data quality cluster is 1%, on average (Supplementary Table S1 of the Supplementary File). Fig. 3 displays the estimated posterior mean for the proportion of the true (unobserved) GS cases that is reported in each Brazilian microregion and state (obtained by averaging the posterior mean of microregions within each state). For microregions belonging to the best data quality group (Cluster 1), the posterior percentage of reported GS cases is 99.01%, on average, which is quite influenced to the mean of the informative prior specified to parameter $\gamma_{1}$ as expected. For areas in Clusters 2, on average, we estimate that more than 90% of the GS cases were correctly registered in SINAN-Syphilis. For Cluster 3 to 6, on average, the proportion of GS cases registered was estimated between 90% and 80%, whereas it was less than 80% for areas belonging to Clusters 7 to 9. In the two worst data quality clusters, our Bayesian model estimated that 34.86% and 30.21% of the GS cases were not reported, respectively. We estimate that all states in the North region registered less than 86.80% of their GS cases. Only four states (Rio Grande do Sul and Parana, in the South region, and São Paulo and Espírito Santo, in Southeast region) reported more than 92.13% of GS cases.

**Fig. 4:**
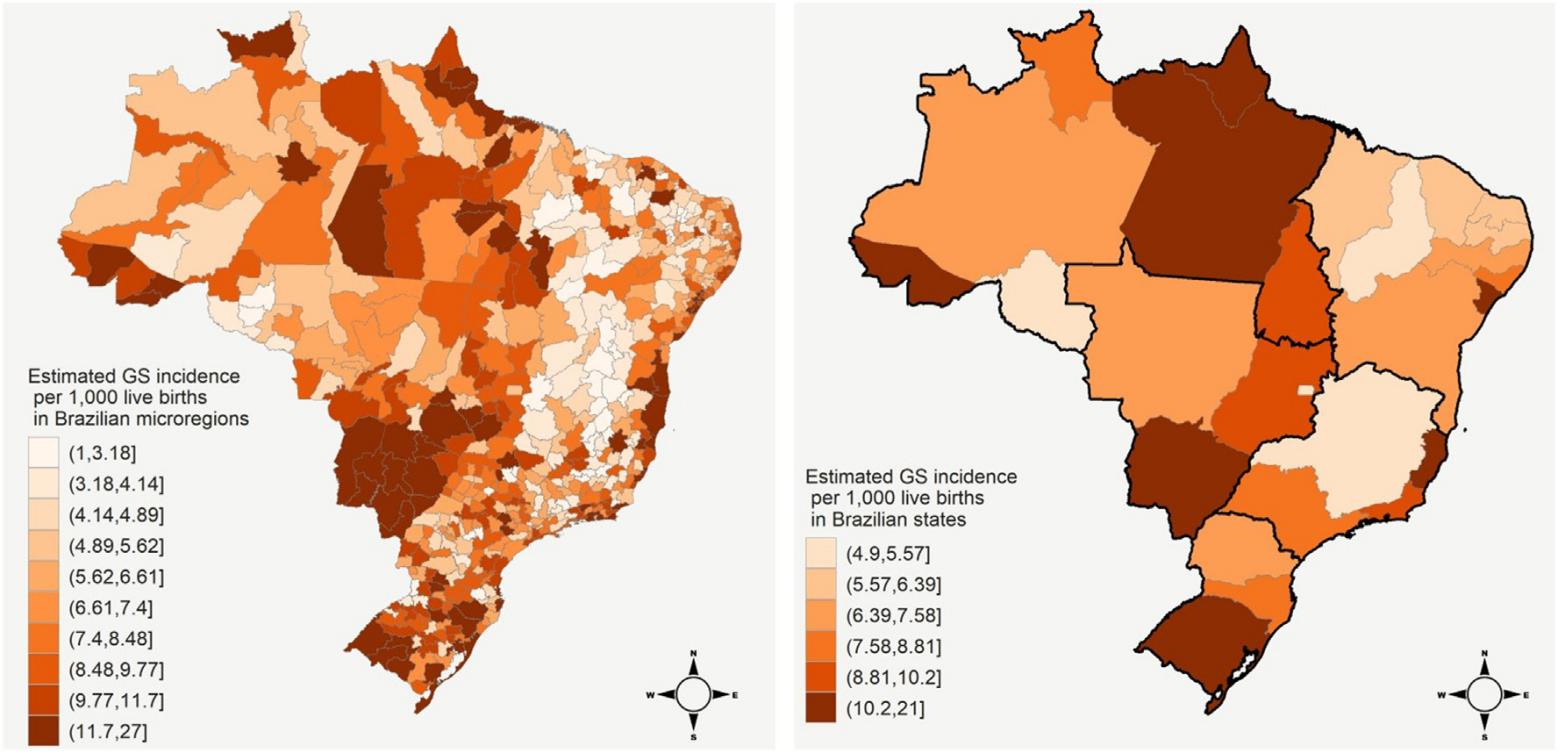
Corrected incidence rates of gestational syphilis (per 1000 live births) in the 557 Brazilian microregions (left) and the 27 Brazilian states (right), 2007–2018, according to the posterior mean of the fitted Bayesian model. Brazilian regions (North, Northeast, Midwest, Southeast and South) are separated by black lines in the right map.

After correcting for underreporting, the GS incidence rate in Brazil was estimated as 10.02 per 1000 live births in the period. We observed a substantial increase in the GS incidence rate for microregions belonging to the worst data quality clusters, which are located, in general, in the Northeast and North regions (Fig. 4). The highest estimated incidence rates at microregion and state levels were, respectively, 26.15 and 20.12, while the lowest ones were around 1.19 and 4.95, respectively. Six Brazilian states presented a posterior mean greater than 10.2 for the GS incidence rate per 1000 live births. Analysis of the effects of covariates considered for modeling the GS incidence rates is provided in Supplementary Table S2 of the Supplementary File.

The mean predicted total GS cases and coverage (percentage of registered cases) of SINAN-Syphilis at state level are shown on Table 2. On average, 45,196 (90%-HPD: 13,299; 79,310) GS cases were missed in Brazil from 2007 to 2018, which represents an estimated detection rate of 87.12% (90%-HPD: 79.40%; 95.83%) of all cases registered at national level. São Paulo (96.41% with 90%-HPD: 93.05%; 99.68%) and Roraima (66.60% with 90%-HPD: 56.01%; 81.83%) were, respectively, the states with the highest and smallest estimated GS coverage.

**Table 2:** Observed cases, posterior mean of predicted number of cases and coverage of gestational syphilis for the 27 Brazilian states, 2007–2018. The 90% highest posterior density interval (90%-HPD) is presented for the predicted quantities.

State	Number of microrregions	Observed cases	Mean predicted underreported cases	Mean predicted total cases	Mean predicted coverage (%)
North region					
Acre (AC)	5	2545	762 (279; 1245)	3307 (2824; 3790)	76.96 (67.15; 90.12)
Amazonas (AM)	13	8592	1681 (525; 2942)	10,273 (9117; 11,534)	83.64 (74.49; 94.24)
Amapá (AP)	4	2026	395 (110; 681)	2421 (2136; 2707)	83.68 (74.84; 94.85)
Pará (PA)	22	14,510	3302 (1148; 5587)	17,812 (15,658; 20,097)	81.46 (72.20; 92.67)
Rondônia (RO)	8	1819	235 (58; 427)	2054 (1877; 2246)	88.56 (80.99; 96.91)
Roraima (RR)	4	937	470 (208; 736)	1407 (1145; 1673)	66.60 (56.01; 81.83)
Tocantins (TO)	8	2577	476 (130; 850)	3053 (2707; 3427)	84.41 (75.20; 95.20)
Northeast region					
Alagoas (AL)	13	4128	1230 (454; 2037)	5358 (4582; 6165)	77.04 (66.96; 90.09)
Bahia (BA)	32	18,885	3904 (1282; 6760)	22,789 (20,167; 25,645)	82.87 (73.64; 93.64)
Ceará (CE)	33	9714	2079 (686; 3596)	11,793 (10,400; 13,310)	82.37 (72.98; 93.40)
Maranhão (MA)	21	7392	1515 (450; 2620)	8907 (7842; 10,012)	82.99 (73.83; 94.26)
Paraíba (PB)	23	4229	822 (224; 1465)	5051 (4453; 5694)	83.73 (74.27; 94.97)
Pernambuco (PE)	18	11,046	2847 (1123; 4878)	13,893 (12,169; 15,924)	79.51 (69.37; 90.77)
Piauí (PI)	15	2812	732 (247; 1255)	3544 (3059; 4067)	79.35 (69.14; 91.93)
Rio Grande do Norte (RN)	19	3217	970 (358; 1631)	4187 (3575; 4848)	76.83 (66.36; 89.99)
Sergipe (SE)	13	3682	1011 (346; 1704)	4693 (4028; 5386)	78.46 (68.36; 91.41)
Midwest region					
Distrito Federal (DF)	1	2447	281 (51; 529)	2728 (2498; 2976)	89.70 (82.22; 97.96)
Goiás (GO)	18	9957	1072 (195; 2052)	11,029 (10,152; 12,009)	90.28 (82.91; 98.08)
Mato Grosso do Sul (MS)	11	10,009	1062 (191; 2034)	11,071 (10,200; 12,043)	90.41 (83.11; 98.13)
Mato Grosso (MT)	22	3998	736 (267; 1236)	4734 (4265; 5234)	84.45 (76.39; 93.74)
Southeast region					
Espírito Santo (ES)	13	8900	1450 (428; 2503)	1035 (9328; 11,403)	85.99 (78.05; 95.41)
Minas Gerais (MG)	66	19,951	3033 (784; 5467)	22,984 (20,735; 25,418)	86.80 (78.49; 96.22)
Rio de Janeiro (RJ)	18	44,910	9113 (2999; 15,153)	54,023 (47,909; 60,063)	83.13 (74.77; 93.74)
São Paulo (SP)	63	63,387	2359 (206; 4735)	65,746 (63,593; 68,122)	96.41 (93.05; 99.68)
South region					
Paraná (PR)	39	14,513	750 (65; 1546)	15,263 (14,578; 16,059)	95.09 (90.37; 99.55)
Rio Grande do Sul (RS)	35	20,153	2077 (378; 4001)	2223 (20,531; 24,154)	90.66 (83.44; 98.16)
Santa Catarina (SC)	20	9422	832 (107; 1640)	10,254 (9529; 11,062)	91.89 (85.17; 98.88)
Brasil (BR)	557	305,758	45,196 (13,299; 79,310)	350,954 (319,057; 385,068)	87.12 (79.40; 95.83)

## Discussion

Our study estimates that around 13% of the notifications of gestational syphilis were not registered in SINAN-Syphilis between 2007 and 2018 in Brazil. The underreporting pattern differs between regions, states and microregions: a higher percentage of underreporting of GS cases was found in the North and Northeast regions, while, in general, in the Southeast region, the observed and corrected cases of GS were similar, considering that the lowest percentage of underreported GS cases were found in the microregions of such region. The lowest estimated coverage of GS cases was found in Roraima State (in North region), while the highest was found in São Paulo (in Southeast region). These different patterns highlight that reporting quality may be related to socio-economic and access to health care services.

To the best of our knowledge, this is the first study mapping the underreporting of GS across the country and estimating the real burden of the disease in all Brazilian microregions and states. A small-scale surveillance study in Minas Gerais State found that only 6.5% of identified GS cases were reported in SINAN-Syphilis between 2007 and 2013.^10^ In Londrina, Paraná State, a study reported that congenital syphilis cases were 50% higher than GS cases from 2009 to 2015,^11^ while Tiago et al. (2017),^9^ using a linkage strategy, identified 46% of GS underreporting in a special indigenous sanitary district in Mato Grosso State. Moreover, Souza et al. (2019)^4^ noted 13.74% of GS under-registration between 2010 and 2013 in the west of São Paulo, the most populous Brazilian state. Comparison of our results with previous studies is not possible considering their heterogeneity in scale, size, and regional focus, as well as methodological differences in the approaches.

We highlight the role of inequalities in access to health services and GS cases detection. Despite the expansion of prenatal coverage in Brazil in the last decade and improvements in actions to reduce GS, the quality of prenatal services is still low in many Brazilian cities, particularly in the North and Northeast regions.^13^ Previous studies also pointed out that the quality of syphilis surveillance in Brazil is intrinsically related to the adequacy of prenatal care,^31^ since it is recommended that the serological test for *T. pallidum* among pregnant women should be requested, at least, at the first prenatal care visit, in the beginning of the third trimester of gestational period, and at the moment of childbirth.^14^ Therefore, inequality in healthcare access contributes directly to underreporting of syphilis cases in the country, particularly among women living in vulnerable contexts, in rural and remote areas, or those facing cultural barriers in access to healthcare services.^13^^,^^15^^,^^16^^,^^31^^,^^37^ Also, the inability of some health professionals to identify syphilis symptoms in the early stage during prenatal care have been associated with underreporting of the disease.

Besides, the access to syphilis tests during the gestational period and the reporting of cases have been directly associated with highest municipal Human Development Index (HDI), most of them located in the Southeast and South region of Brazil.^13^^,^^31^^,^^32^^,^^37^ The availability of syphilis rapid test in healthcare service, the timely access to results of diagnostic tests, in addition to postponing the search for test results due to the absence of symptoms in some stages of the infection contribute to GS underreporting. Therefore, the highest quality of prenatal care services is closely related to access to syphilis tests, and could contribute to reduce failures in reporting GS.^13^^,^^31^^,^^32^^,^^37^ Aligned with our results, we found that the highest incidence rates of underreported cases of GS occurred in North and Northeast, regions that concentrated the microregions with the highest deprivation levels, and with less coverage and access to diagnostic test and prenatal care, factors previously associated with underreporting and the GS incidence rate in Brazil.^31^^,^^32^^,^^37^

The increasing incidence of GS represents a growing concern for health professionals and public health in Brazil,^4^^,^^14^ and failure to correctly diagnose syphilis during pregnancy contributes to non-treatment, since the disease could be asymptomatic, or lead to inadequate adherence to treatment, particularly when the diagnosis occurs at the end of the gestational period. Additionally, stigma and fear of discrimination may discourage pregnant individuals from seeking care for treatment, further contributing to the inadequate management of syphilis during pregnancy, which increases the occurrence of adverse health outcomes for pregnant individuals. Also, failures in the diagnosis of GS could increase the vertical transmission of syphilis, as well as it enhances the chance of adverse effects on the newborn.^6^^,^^38^ Moreover, it is important to highlight that syphilis should be considered during pregnancy as a fetal emergency due to the high proportion of early or late fetal deaths related to lack of treatment.^14^^,^^39^ Thus, underreporting of GS can occult the real epidemiological profile of the disease in the country and reduce the possibility of achieving the goals of the World Health Organization regarding GS and congenital syphilis.^40^ Besides, it is important highlighting that the variation of GS reporting coverage identified across Brazilian microregions and states expresses differences in capabilities and human resources of health systems, particularly related to prenatal and childbirth care across the country. This scenario creates the urgency of actions and programs for each region to improve GS diagnosis and registration of cases in the SINAN-Syphilis.

### Strengths and limitations

The study is a first attempt to estimate the underreporting of GS in Brazil and correct incidence rates for the whole country, which could help to address the country’s gap of knowledge regarding GS notification. We provide robust subsidies to Brazilian health authorities to better increase surveillance for GS across the country, highlighting municipalities where improvements in surveillance and case reporting should be prioritized. Our results could allow policymakers and stakeholders to improve policy and action to reduce GS, and, therefore, contribute to reducing mother-to-child transmission and the adverse effects on birth outcomes related to congenital syphilis. Also, it could be used by the Brazilian Ministry of Health in planning the *Previne Brasil*, a public policy that will transfer financial resources to municipalities based on their health indicators, such as the proportion of pregnant with at least six prenatal care visits and tested for syphilis.^41^

Despite the relevance of our results, the study presented some limitations. First, the percentages of underreported cases were calculated for the entire study period, not allowing for analysis of possible improvements or setbacks in the quality of information over time. Second, the model imposes the same reporting probability for areas within the same data quality cluster. However, we found this is the best methodology to be applied, given the restricted type of prior information we have available on underreporting of GS in Brazil.

### Conclusion

Brazilian microregions and states in the North and Northeast regions presented the highest values of underreporting of GS, which directly affects the knowledge of the epidemiological profile and the estimation of the social, economic, and health burdens associated with the disease. Conversely, microregions and states in the Southern region presented the lowest values of underreported cases. This scenario highlights the regional disparities in the incidence rate of GS and the quality of prenatal care in the Brazilian context, as well as emphasizes the need to improve the quality of information registered in SINAN-Syphilis, considering the importance of disease surveillance across the country. Furthermore, strategies to prevent, control, and maintain the surveillance of GS based only on the data made available in the national health information system without correcting for underreported cases can lead to misguided actions and health policies, particularly related to health finance and the distribution of human resources across the regions.

This study provides robust evidence that can be used to enhance national surveillance system and particularly specific policies for GS detection, early treatment and disease control, based in prior information and using a statistical methodology that could be applied by health manager of many countries.

## Contributors

G.L.O., M.Y.I. and A.J.F.F. conceptualized this study and contributed to its design.

G.L.O. conducted the statistical analysis.

G.L.O., M.Y.I., A.J.F.F., C.A.S.S.T., R.A., R.F., R.L., M.P., E.S.P., and A.M.C. contributed to the result analysis and interpretation.

G.L.O., M.Y.I., A.J.F.F., C.A.S.S.T., I.O.S and M.A.S performed literature review.

G.L.O. and A.J.F.F. formatted tables, figures and wrote the first draft.

M.Y.I. and M.L.B acquired the data, funding and coordinated the study.

G.L.O., A.J.F.F., C.A.S.S.T. and M.Y.I. have accessed and verified the underlying data.

G.L.O., A.J.F.F., E.S.P., M.Y.I. and M.L.B were responsible for the decision to submit the manuscript.

All authors have access to all the data reported in the study, wrote the manuscript and approved the final version.

## Data sharing statement

The GS data are publicly available from the Brazilian National Notifiable Diseases Information System (SINAN) at http://portalsinan.saude.gov.br/sinan-net. Additionally, all data collected for the study and posterior results are available from the corresponding author upon reasonable request.

## Editorial disclaimer

The Lancet Group takes a neutral position with respect to territorial claims in published maps and institutional affiliations.

The abstract translated to Portuguese was submitted by the authors and we reproduce it as supplied. It has not been peer reviewed. Our editorial processes have only been applied to the original abstract in English, which should serve as a reference for this manuscript.

## Declaration of interests

We declare no competing interests.

## Appendix ASupplementary data

Translated Summary_Portuguese R2
