# Supplementary material for: Estimating the real burden of gestational syphilis in Brazil, 2007–2018: a Bayesian modeling study
Source: Lancet Reg Health Am. 2023 Aug 1;25:100564. doi: 10.1016/j.lana.2023.100564 (PMC10415804; doi:10.1016/j.lana.2023.100564)
Supplement: Translated Summary_Portuguese R2 [file mmc1.docx]

**Estimating the real burden of gestational syphilis in Brazil, 2007 to 2018: a Bayesian modeling study**

**Estimando a real magnitude da sífilis gestacional no Brasil, 2007 a 2018: um estudo baseado em modelagem Bayesiana**

**Resumo**

**Introdução:** Embora vários estudos tenham estimado a incidência de sífilis gestacional (SG) em vários países, a correção da subnotificação raramente é considerada. Este estudo teve como objetivo estimar o nível de sub-registro e corrigir as taxas de incidência da SG nas 557 microrregiões brasileiras.

**Métodos:** As notificações brasileiras de SG entre 2007 e 2018 foram obtidas do sistema SINAN-Sífilis. Foi realizada uma análise de cluster para agrupar as microrregiões de acordo com a qualidade da notificação de SG. Um modelo de regressão Poisson Bayesiano hierárquico foi aplicado para estimar as probabilidades de notificação entre os clusters e corrigir as taxas de incidência associadas.

**Resultados:** Estimou-se que 45.196 (90%-HPD: 13.299; 79.310) casos de SG foram subnotificados no Brasil de 2007 a 2018, representando uma cobertura de 87,12% (90%-HPD: 79,40%; 95,83 %) de casos registrados, onde HPD representa o intervalo de credibilidade Bayesiano de maior densidade *a posteriori*. O nível de subnotificação difere ao longo do país, com as microrregiões das regiões Norte e Nordeste apresentando o maior percentual de casos perdidos. Após a correção da subnotificação, a estimativa da taxa de incidência de SG no Brasil aumentou de 8,74 para 10,02 por 1.000 nascidos vivos no período.

**Interpretação:** Nossos achados destacam disparidades no nível de registro e na taxa de incidência da SG no Brasil, refletindo a heterogeneidade regional na qualidade da vigilância da sífilis, acesso ao pré-natal e serviços de assistência ao parto. Este estudo fornece evidências robustas para aprimorar os sistemas nacionais de vigilância e orientar políticas específicas para o controle e detecção da SG, além de potencialmente mitigar as consequências prejudiciais da transmissão de mãe para filho. A metodologia utilizada pode ser aplicada em outras regiões para corrigir a subnotificação de doenças.

**Financiamento:** Conselho Nacional de Desenvolvimento Científico e Tecnológico (CNPq-Brasil); The Gates Foundation; and Wellcome Trust.

**Keywords:** Correção de subnotificação; Infecção Sexualmente Transmissível; Sífilis; Sífilis durante a gravidez; Sub-registro.
