# Supplementary material for: Estimating the real burden of gestational syphilis in Brazil, 2007–2018: a Bayesian modeling study
Source: Lancet Reg Health Am. 2023 Aug 1;25:100564. doi: 10.1016/j.lana.2023.100564 (PMC10415804; doi:10.1016/j.lana.2023.100564)
Supplement: Supplementary file [file mmc2.pdf]

Supplementary File to "Estimating the real burden of gestational syphilis in Brazil, 2007 to 2018: a Bayesian modeling study"

Guilherme Lopes de Oliveira[a,b,*], Andrêa JF Ferreira[a,c], Carlos Antônio de S.S. Teles[a], Enny S. Paixao[a,d], Rosemeire Fiaccone[a,e], Raquel Lana[a,f], Rosana Aquino[a,g], Andrey Moreira Cardoso[h], Maria Auxiliadora Soares[a,g], Idália Oliveira dos Santos[a,g], Marcos Pereira[a,g], Maurício L. Barreto[a,g] and Maria Yury Ichihara[a]

[a] *Centre of Data and Knowledge Integration for Health (CIDACS), Instituto GonÃ§alo Moniz, Fiocruz, Bahia, Brazil*
[b] *Departament of Computing, Federal Centre of Technological Education of Minas Gerais, Minas Gerais, Brazil*
[c] *The Ubuntu Center on Racism, Global Movement, Population and Equity, School of Public Health, Drexel University, Pennsylvania, US*
[d] *London School of Hygiene and Tropical Medicine, London, UK*
[e] *Institute of Mathematics, Statistics Department, Federal University of Bahia, Bahia, Brazil*
[f] *Barcelona Supercomputing Center, Catalonia, Spain*
[g] *Institute of Collective Health, Federal University of Bahia, Bahia, Brazil*
[h] *National School of Public Health, Fiocruz, Rio de Janeiro, Brazil.*

1. Bayesian statistical model specification

We define Y_i as the total reported (observed) gestational syphilis (GS) cases in microregion i, $i = 1, ..., 557$ and following Oliveira *et al.* (2022)[1] we assume that

$$Y_i|\mu_i, \epsilon_i \overset{ind}{\sim} \text{Poisson}\left(\mu_i \epsilon_i\right), \tag{1}$$

where $\mu_i > 0$ and $0 < \epsilon_i < 1$ denote, respectively, the GS incidence rate and the proportion of the true (unobserved) cases reported in the i-th microregion. In more theoretical terms, the model presented in Equation (1) for the observed cases Y_i is derived from a scenario where the true cases T_i are modeled as $T_i|\mu_i \sim Poisson(\mu_i)$ and then the observed cases are obtained from binomial sampling as $Y_i|T_i, \epsilon_i \sim Binomial(T_i, \epsilon_i)$. In this context, it can be proved that, after marginalizing the joint model obtained from these two equations with respect to T_i (this is needed since T_i is not available), the model for the observed cases Y_i is given by $Y_i|\mu_i, \epsilon_i \sim Poisson(\mu_i \epsilon_i)$. Therefore, μ_i is the GS incidence rate related to the true total GS cases T_i from which only a proportion ϵ_i is observed and represented by Y_i.

When considering the Poisson model shown in Equation (1), special attention must be given do the modeling of parameter ϵ_i because this model suffers from the lack of identifiability. This occurs because only the product $\eta_i = \epsilon_i \mu_i$ is identified from the observed data since any other parameter combination, say $\tilde{\mu}_i$ and $\tilde{\epsilon}_i$ would provide the same likelihood function. Such a problem can be overcome through the introduction of additional information to differentiate between parameters μ_i and ϵ_i. In practice, it is more common and feasible to include additional information about the reporting process, represented by parameter ϵ_i, then about the occurrence process, represented by parameter μ_i. Such extra information can be provided, for instance, by validation datasets, active search surveys or experts' opinion. The source of information to be used in a specific application will depend on which one is available.

To overcome the identifiability issue in our application to GS Brazilian datasets, we follow the Bayesian approach of Oliveira *et al.* (2022).[1] Provided that the areas are grouped into K data quality clusters, their model assumes that

$$\epsilon_i = 1 - \mathbf{G}_i' \boldsymbol{\gamma}, \tag{2}$$

where $G_i = (G_{i1}, \ldots, G_{iK})$ is a vector of length K composed by binary inputs ($G_{ij} = 0$ or $G_{ij} = 1$ for all i, j) responsible for identifying the data quality group to which area i belongs and $\boldsymbol{\gamma} = (\gamma_1, \gamma_2, \ldots, \gamma_K)$ is a K-dimensional parameter

guilhermeoliveira@cefetmg.br (G.L.d. Oliveira)
ORCID(s): 0000-0003-3220-6356 (G.L.d. Oliveira)

vector from which is possible to obtain the underreporting probabilities throughout the hierarchical clustering structure, with $0 \leq \gamma_j \leq 1$ for all $j = 1, \ldots, K$. As proposed in Oliveira *et al.* (2022),[1] the clusters must be labeled in such a way that $\boldsymbol{G}_i = (1, 0, \ldots, 0)$ for all areas belonging to the best data quality group, where $\epsilon_i = 1 - \gamma_1$; $\boldsymbol{G}_i = (1, 1, \ldots, 0)$ for all areas belonging to the second best data quality group, where $\epsilon_i = 1 - \gamma_1 - \gamma_2$; and so on, implying that all areas within the worse data quality cluster are associated to $\boldsymbol{G}_i = (1, 1, \ldots, 1)$ in which $\epsilon_i = 1 - \gamma_1 - \cdots - \gamma_K$. Parameters in $\boldsymbol{\gamma}$ have an appealing practical interpretation: γ_1 represents the proportion of underreported cases in areas classified in the highest level of data quality; γ_2 is the increment on such proportion for areas experiencing the second highest data quality level, and so on.

In the practical sense, the most important and attractive feature of this modeling strategy is that, to attain identifiability, it only requires an informative prior distribution for parameter γ_1. An informative prior means a distribution that is highly concentrated in a specific subset of the parametric space. This parameter represents the proportion of underreporting in those areas belonging to the best data quality cluster. In our application to GS data in Brazil, with basis on information collected from experts' on the study of syphilis burden in Brazil, we performed a sensitivity analysis considering four different prior distributions for γ_1, namely, we assume that γ_1 $Uniform(0, 0.02)$, γ_1 $Uniform(0, 0.05)$, γ_1 $Uniform(0.05, 0.10)$ and γ_1 $Uniform(0, 0.10)$. Respectively, these priors imply that areas classified in the highest level of data quality report, in average, 99%, 97.5%, 95% and 92.5% of their total of GS cases. All of them impose that less than 10% of the true GS cases are missed in such areas. For all other groups of areas, the estimation of the reporting probability is completely guided by the information contained in the data and in the hierarchical structure of the joint prior distribution elicited to parameter vector $\boldsymbol{\gamma} = (\gamma_1, \gamma_2, \ldots, \gamma_K)$, which is given by the following Conditional Uniform distribution:

$$\begin{cases} \gamma_1 & \sim & \mathcal{U}(a_1, b_1), \\ \gamma_2 \mid \gamma_1 & \sim & \mathcal{U}(0, 1 - \gamma_1), \\ \gamma_3 \mid \gamma_1, \gamma_2 & \sim & \mathcal{U}(0, [1 - \gamma_1 - \gamma_2]), \\ \vdots & & \\ \gamma_K \mid \boldsymbol{\gamma}_{1:K-1} & \sim & \mathcal{U}\left(0, [1 - \sum_{j=1}^{K-1} \gamma_j]\right), \end{cases} \tag{3}$$

where hyperparameters $0 < a_1 < b_1 < 1$ must be chosen guided by experts' opinion in order to provide an informative (low variance) prior to γ_1. Thus, each $\gamma_j,\ j = 2, \ldots, K$, has a completely non-informative prior distribution given the previous parameters $\gamma_1, \ldots, \gamma_{j-1}$. The hierarchical structure of prior in Equation (3) ensures that $0 < \sum_{j=1}^{K} \gamma_j < 1$, that is, all $\epsilon_i,\ i = 1, \ldots, 557$, given in Equation (2) represents a (reporting) probability between 0 and 1. The hierarchical prior distribution shown in Equation (3) is a particular case of the Generalized Beta prior distribution, which allows for density shapes different from uniform within each cluster (for details see Oliveira *et al.* (2022)[1]).

With respect to the GS incidence rate modeling, we assume the following regression structure

$$log(\mu_i) = log(P_i) + \beta_0 + \beta_1 x_{1i} + \beta_2 x_{2i} + \beta_3 x_{3i} + \phi_i + \delta_i, \tag{4}$$

where P_i= total live births (offset), x_{1i} = Brazilian deprivation index, x_{1i} = proportion of live births with adequate prenatal care, x_{1i}= proportion of non-white live births (see variables description in Table 1 of the main manuscript). Variables x_1 ,x_2 and x_3 were standardized before applying the model, that is, each of them was centered with relation to its observed mean and divided by its observed standard deviation. Terms ϕ_i and δ_i are, respectively, random effects accounting for residual overdispersion induced by local and spatial variations.

Since after standardization each covariate x_1 to x_3 becomes centered zero, then β_0 is interpreted as the mean reported number of GS cases, on the logarithmic scale. To avoid the generation of unrealistic values for the mean GS incidence rate (especially quite elevated values), we assume a prior distribution $N(-5, 1)$ for β_0 to represent our belief that is not plausible a very high value (such as over 1 million) for the total number of new leprosy cases at the mean level. For all remaining regression parameters, β_0 and $\beta_1, \ldots, \beta_3$, we elicit a non-informative prior distribution $N(0, 100)$, where $N(\theta, \sigma^2)$ represents a Normal/Gaussian distribution with mean θ and variance σ^2.

The model specification is completed with the following prior distributions for the random effects and their precision terms. As it is usual in the literature, the prior distribution for the spatially structured random effect ϕ_i, with precision parameter ν, is represented by an intrinsic conditional auto-regressive (ICAR) model.[2] Here, a neighbor of an area i was defined as any $i' \neq i$ sharing a geographical boundary with i. The unstructured local effect δ_i is

assumed to follow a Normal prior distribution $N(0, \sigma_\delta)$. For each of the precision parameters ν and $1/\sigma_\delta^2$ we elicited a $N(0,1)$ truncated at 0 to assume only positive values.

For each microregion, provided that μ_i and ϵ_i are estimated, the underreported GS cases, denoted by Z_i, can be obtained from the predictive posterior distribution

$$Z_i|\mu_i, \epsilon_i \overset{ind}{\sim} \text{Poisson}\left(\mu_i(1-\epsilon_i)\right). \tag{5}$$

1.1. Model implementation and validation

The model was implemented using the NIMBLE[3] package from software R.[4] The Markov chain Monte Carlo (MCMC) scheme, four chains were used, each of them running a total of 450,000 iterations. The initial 150,000 iterations were discarded as burn-in period and a lag of 250 iterations was considered to avoid correlated posterior samples. Thus, the analyzes were based on 4,800 posterior samples for each model parameter. Trace plots of the MCMC samples were used to inspect convergence, and the potential scale reduction factor (PSRF), proposed by Brooks and Gelman (1998)[5] was also computed. We used the geobr[6] package (MIT license https://ipeagit.github.io/geobr/) from software R to create the maps produced in this work in order to visualize the spatial analysis. All data considered in the statistical analysis are public available and can be accessed in the Supplementary Material.

By varying the number of clusters K and the prior distribution for parameter γ_1 a total of twelve different models were fitted. The WAIC[7] and the LPML[8] model evaluation statistics were considered for model selection. For the WAIC, the smaller the value, the better the model fitted the data. Larger values of LPML indicate better fit.

2. Results

2.1. Sensitivity analysis (model choice)

To estimate the number of data quality clusters, we analyzed the within cluster sums of squares and the elbow method indicated that around K=10 is a good choice for the number of clusters (see Figure 2 of the main manuscript). A sensitivity analysis including K=9, K=10 and K=11 was performed. Along with the number of clusters, we also varied the prior distributions for γ_1, considering Uniform$(0, 0.02)$, Uniform$(0, 0.05)$, Uniform$(0.05, 0.10)$ and Uniform$(0, 0.10)$. Clusters were hierarchically related from the best to the worst with respect to data quality. For doing so, we analyzed the within-cluster centroid containing the mean value for the seven variables used in the clustering analysis. The higher the means of the variables, the better the data quality.

According to some model evaluation measures presented in Table 1 below, for the GS database the best fitted model consider K=9 data quality clusters and that, *a priori*, the proportion of missed GS cases in the best cluster is around 1%, which is imposed by the informative prior distribution γ_1 $Uniform(0, 0.02)$. This model presented the lowest value for the WAIC and the highest LPML measure. For the WAIC, the smaller the value, the better the model fitted the data. Larger values of LPML indicate better fit.

Table 1
Evaluation metrics for adjusted models (sensitivity analysis). The best model is highlighted in each case. WAIC=Watanabe-Akaike information criterion;[7] LPML= logarithm of the Pseudo-marginal likelihood;[8] PSRF=potential scale reduction factor.[5]

Number of Clusters	Prior to γ_1	WAIC	LPML	Multivariate PSRF
**K=9**	**U(0,0.02)**	**4,919.14**	**-2,694.11**	**1.09**
K=10	U(0,0.02)	4,920.17	-2,706.18	1.08
K=11	U(0,0.02)	4,919.49	-2,694.16	1.05
K=9	U(0,0.05)	4,920.78	-2,696.62	1.08
K=10	U(0,0.05)	4,919.95	-2,700.89	1.05
K=11	U(0,0.05)	4,919.88	-2,711.19	1.07
K=9	U(0,0.10)	4,919.23	-2,701.85	1.06
K=10	U(0,0.10)	4,919.54	-2,703.15	1.08
K=11	U(0,0.10)	4,920.86	-2,708.59	1.05
K=9	U(0.05,0.10)	4,919.69	-2,704.25	1.12
K=10	U(0.05,0.10)	4,919.85	-2,698.37	1.09
K=11	U(0.05,0.10)	4,920.03	-2,705.97	1.12

2.2. Posterior summaries for all model parameters

Considering the best-fitted model identified from the sensitivity analyses, we mapped the posterior mean for the incidence rates and the reporting probabilities of GS cases at microregion and state levels. The PSRF[5] was less than 1.05 for all regression coefficients and precision parameters and a multivariate PSRF of 1.09, which is sufficient to indicate convergence to the target posterior distribution.

For each microregion, we generate the missed GS cases from Equation (5) using each of the 4,800 posterior samples of the model parameters. We calculated the mean of the posterior predictive distribution and the associated 90%-HPD. Results at state level are provided in Table 2 of the main manuscript.

Posterior mean (Mean), the posterior standard deviation (SD) and associated with 90% highest posterior density intervals (90%-HPD) are provided in Table 2 below. Significance of covariate effects is considered when the associated 90%-HPD does not contain the value 0. We calculated the incidence rate ratio (IRR) for Poisson parameters with their respective 90%-HPD intervals. The IRR indicates the effect in the mean incidence when a unity change occurs in the associated standardized explanatory variable.

Table 2 provides estimated effects for the three covariates considered in Equation (4) for modeling the GS indicidence rate: the Brazilian deprivation index, the proportion of live births with less than 7 prenatal consultations and the proportion of non-white live births. They were statistically significant at level 10% since the 90%-HPD intervals associated to their regression coefficients do not contain the value 0. It is important noticing that covariate BDI presented a "protective effect", that is, a negative regression coefficient. It would be explained by the fact that, in more deprived areas the level of underreporting is higher. Although the correction of cases in such are is the highest, as the absolute number of cases are small, the incidence rates are estimated in lower values resulting in a negative association with the BDI.

Table 2
Posterior summaries for the regression effects $\boldsymbol{\beta}$ and $\boldsymbol{\gamma}$ and the model variance parameters; Brazilian Gestational Syphilis data 2007-2018. For each parameter we provide the posterior mean (Mean), the posterior standard deviation (SD) and the 90% highest posterior density (90%-HPD) interval. The incidence rate ratio (IRR) for Poisson parameters are also provided, along with the associated 90%-HPD interval.

Incidence rate	Parameter description	Mean	SD	90%-HPD	IRR (90%-HPD)
β_0	incidence rate intercept	-4.981	0.042	(-5.050, -4.912)	-
β_1	inadequacy of prenatal care	0.254	0.047	(0.176, 0.329)	1.291 (1.187, 1.386)
β_2	proportion of non-white live births	0.311	0.068	(0.207, 0.429)	1.368 (1.218, 1.527)
β_3	Brazilian deprivation index	-0.429	0.052	(-0.513, -0.342)	0.652 (0.598, 0.710)
σ^2_ϕ	spatial effect variance parameter	0.304	0.068	(0.194, 0.412)	-
σ^2_δ	unstructured effect variance parameter	0.092	0.016	(0.065, 0.118)	-
Reporting probability	Parameter description	Mean	SD	90%-HPD	-
ϵ_1	reporting level for areas in Cluster 1	0.990	0.006	(0.980, 0.998)	-
ϵ_2	reporting level for areas in Cluster 2	0.917	0.042	(0.852, 0.985)	-
ϵ_3	reporting level for areas in Cluster 3	0.899	0.042	(0.832, 0.968)	-
ϵ_4	reporting level for areas in Cluster 4	0.867	0.047	(0.789, 0.946)	-
ϵ_5	reporting level for areas in Cluster 5	0.848	0.048	(0.771, 0.930)	-
ϵ_6	reporting level for areas in Cluster 6	0.831	0.049	(0.755, 0.913)	-
ϵ_7	reporting level for areas in Cluster 7	0.766	0.058	(0.672, 0.860)	-
ϵ_8	reporting level for areas in Cluster 8	0.698	0.058	(0.599, 0.791)	-
ϵ_9	reporting level for areas in Cluster 9	0.651	0.062	(0.552, 0.757)	-

References

[1] Oliveira GL de, Argiento R, Loschi RH, AssunÃ§Ã£o RM, Ruggeri F, Branco MD. (2022). Bias Correction in Clustered Underreported Data. *Bayesian Analysis* , 17:95–126.

[2] Besag, J, York, J, Mollié, A. (1991). Bayesian image restoration, with two applications in spatial statistics. *Annals of the Institute of Statistical Mathematics*, 43(1), 1—20.

[3] de Valpine P, Turek D, Paciorek CJ, Bergman CA, Lang DT, Bodik R. (2017). Programming with models: writing statistical algorithms for general model structures with NIMBLE. *Journal of Computational and Graphical Statistics*, 26(2):403–13.

[4] R Core Team. (2021). R: a language and environment for statistical computing. *R CRAN*, Available at: https://www.R-project.org/ (accessed Sept 6, 2022).

[5] Brooks SP, Gelman A. (1998). General methods for monitoring convergence of iterative simulations. *Journal of computational and graphical statistics*, 7(4):434–55.

[6] Pereira RH, GonÃ§alves CN. (2019). geobr: Loads Shapefiles of Official Spatial Data Sets of Brazil. *GitHub repository*. Available at: https://ipeagit.github.io/geobr/

[7] Watanabe, S. (2010). Asymptotic Equivalence of Bayes Cross Validation and Widely Applicable Information Criterion in Singular Learning Theory. *Journal of Machine Learning Research*, 11:3571–3594.

[8] Ibrahim, J. G., Chen, M-H., and Sinha, D. (2001). Bayesian Survival Analysis. *New York: Springer-Verlag*, p.p. 589.
